# Epigenetic regulation of *MDR1* gene through post-translational histone modifications in prostate cancer

**DOI:** 10.1186/1471-2164-14-898

**Published:** 2013-12-17

**Authors:** Rui Henrique, Ana Isabel Oliveira, Vera L Costa, Tiago Baptista, Ana Teresa Martins, António Morais, Jorge Oliveira, Carmen Jerónimo

**Affiliations:** 1 Cancer Epigenetics Group, Research Center of the Portuguese Oncology Institute, Porto, Rua Dr. António Bernardino de Almeida, 4200-072 Porto, Portugal; 2 Department of Pathology, Portuguese Oncology Institute, Porto, Rua Dr. António Bernardino de Almeida, 4200-072 Porto, Portugal; 3 Department of Urology, Portuguese Oncology Institute, Porto, Rua Dr. António Bernardino de Almeida, 4200-072 Porto, Portugal; 4 Department of Pathology and Molecular Immunology, Institute of Biomedical Sciences Abel Salazar, University of Porto, Largo Prof. Abel Salazar 2, 4099-003 Porto, Portugal

**Keywords:** CpG island hypermethylation, Epigenetic regulation, Histone post-translational activation/repression marks, *MDR1*, P-gp, Prostate

## Abstract

**Background:**

Multidrug resistance 1 (*MDR1*) gene encodes for an ATP binding cassette transporter - P-glycoprotein (P-gp) - involved in chemoresistance to taxanes. *MDR1* promoter methylation is frequent in prostate carcinoma (PCa), suggesting an epigenetic regulation but no functional correlation has been established. We aimed to elucidate the epigenetic mechanisms involved in *MDR1* deregulation in PCa.

**Results:**

*MDR1* promoter methylation and P-gp expression were assessed in 121 PCa, 39 high-grade prostatic intraepithelial neoplasia (HGPIN), 28 benign prostatic hyperplasia (BPH) and 10 morphologically normal prostate tissue (NPT) samples, using quantitative methylation specific PCR and immunohistochemistry, respectively. PCa cell lines were exposed to a DNA methyltransferases inhibitor 5-aza-2′deoxycytidine (DAC) and histone deacetylases inhibitor trichostatin A (TSA). Methylation and histone posttranscriptional modifications status were characterized and correlated with mRNA and protein expression. *MDR1* promoter methylation levels and frequency significantly increased from NPTs, to HGPIN and to PCa. Conversely, decreased or absent P-gp immunoexpression was observed in HGPIN and PCa, inversely correlating with methylation levels. Exposure to DAC alone did not alter significantly methylation levels, although increased expression was apparent. However, P-gp mRNA and protein re-expression were higher in cell lines exposed to TSA alone or combined with DAC. Accordingly, histone active marks H3Ac, H3K4me2, H3K4me3, H3K9Ac, and H4Ac were increased at the *MDR1* promoter after exposure to TSA alone or combined with DAC.

**Conclusion:**

Our data suggests that, in prostate carcinogenesis, *MDR1* downregulation is mainly due to histone post-translational modifications. This occurs concomitantly with aberrant promoter methylation, substantiating the association with P-gp decreased expression.

## Background

P-glycoprotein (P-gp), a membrane protein that acts as an ATP-binding cassette (ABC) transporter, is actively involved in the efflux of antineoplastic agents from cancer cells [1,2]. Together with the other members of the ABC transporters family, it provides protection against xenobiotics and certain endogenous molecules, producing the multidrug resistance (MDR) phenotype, by which cancer cells become insensitive or unresponsive to a wide spectrum of drugs [3]. This transporter is encoded by the *MDR1/ABCB1* gene (*multidrug resistance receptor 1*/ *ATP-binding cassette, sub-family B, member 1*), mapped at 7q21, which is usually expressed in a limited number of tissues (gastrointestinal, liver, kidney and capillary endothelial cells in brain, ovary, and testis) [4]. The *MDR1* gene is composed by two promoters, a major downstream/proximal (DSP) and a minor upstream (USP), along with 28 exons [4-7]. In human cells, the DSP, which encompasses one CpG island, along with two other CpG islands (one located in exon 1 and the other in intron 1) regulates most of the transcriptional activity [4,6-8]. Like other promoters, sequences downstream of the initiation site are also important for the overall transcription regulation [9] and it has been shown that *MDR1* transcription might be modulated by proteins capable of modifying nucleosomal histones [10]. Thus, epigenetic mechanisms are likely to play an important role in *MDR1* expression regulation.

Remarkably, *MDR1* promoter methylation is very frequent in prostate carcinoma (PCa) [11-13], which represents the second most frequent neoplasia among male population worldwide (13.6% of the total) and the fifth most common cancer overall [14], being the second leading cause of cancer-related death in men [15]. This observation, in conjunction with the significantly lower levels of methylation observed in non-tumorous prostate tissues, has placed *MDR1* in the restricted group of candidate epigenetic-based biomarkers specific for PCa [16]. Because P-gp expression has been found to be generally lower in PCa than normal prostate glands [17,18], cancer-associated aberrant promoter methylation has been postulated as the main mechanism underlying *MDR1* silencing in PCa [11,12]. However, functional evidence for that association has not been reported yet.

It is widely acknowledged that DNA methylation and other epigenetic mechanisms, such as histone modifications, act in concert to regulate gene expression through alterations in chromatin structure [19,20]. Aberrant methylation of promoter CpG islands results in transcriptional silencing through several mechanisms, including the attraction of proteins that interact with histone deacetylases, and chromatin condensation, precluding the binding of transcriptional factors to the promoter, thus modulating gene expression and, consequently, tumour phenotype. As a result, the bulk of methylation in a tumour may reflect its biological and clinical behavior [19,21]. Likewise, histone post-translational modifications are also strongly correlated with transcription regulation. Both positive (H3Ac; H3K4me2; H3K4me3; H3K9Ac; H4Ac) and negative-acting marks (H3K9me3; H3K27me3) are established across promoters during gene activation or gene repression, respectively, and the interplay of those histone modifications ultimately control gene expression [22]. Importantly, the interplay between DNA methylation and histone modifications during gene silencing is currently acknowledged, as well as their importance in the integration of environmental and intrinsic stimuli in gene expression control.

Thus, we aimed to elucidate the role of epigenetic mechanisms in *MDR1* deregulation in prostate carcinogenesis. For that purpose, *MDR1* promoter methylation and P-gp expression was firstly assessed in a series of PCa, high grade prostatic intraepithelial neoplasia (HGPIN) – a precursor lesion of PCa – and non-tumorous prostate tissues [benign prostatic hyperplasia (BPH) and morphologically normal prostate tissue (NPT)]. Then, PCa cell lines were exposed to epigenetic modulating drugs and their effect on *MDR1* promoter methylation and mRNA and protein expression was assessed. Finally, activating histone post-translational modifications associated with the *MDR1* promoter region, prior and after exposure to epigenetic modulating drugs, were surveyed and correlated with gene expression status.

## Results

### Clinical and pathological characteristics

The clinical and pathological characteristics of the patients enrolled in this study are illustrated in Table 1. As expected, PSA levels were higher in patients with PCa, but a significant overlap with BPH cases was apparent (Mann–Whitney, p = 0.002). Statistically significant differences in patient’s age were detected among the three groups of patients (Kruskall-Wallis, p = 0.002). Significant differences were disclosed only between the median age of BPH and PCa patients (Mann–Whitney, Bonferroni-adjusted, p = 0.001).

**Table 1:** Clinical and pathological characteristics of patient populations

	**NPT**	**BPH**	**HGPIN**	**PCa**
Number of patients	10	26	37	121
Age, median (range)	60 (50–80)	68 (54–79)	65 (40–74)	64 (40–74)
PSA, ng/mL, median (range)	n.av.	5.8 (0.8-32.5)	8.03 (3.35-16.9)	9.3 (3.1-48.3)
Gleason score, median (range)	n.a.	n.a.	n.a.	7 (4–9)
Pathological stage (%)				
pT2	n.a.	n.a.	n.a.	63 (52.1%)
pT3	n.a.	n.a.	n.a.	58 (47.9%)

NPT - morphologically normal prostate tissue, BPH - benign prostatic hyperplasia, HGPIN - high grade prostatic intraepithelial neoplasia, PCa - prostate carcinoma, n.av. not available and n.a. - not applicable.

### MDR1 promoter methylation in prostatic tissue

**Figure 1:**
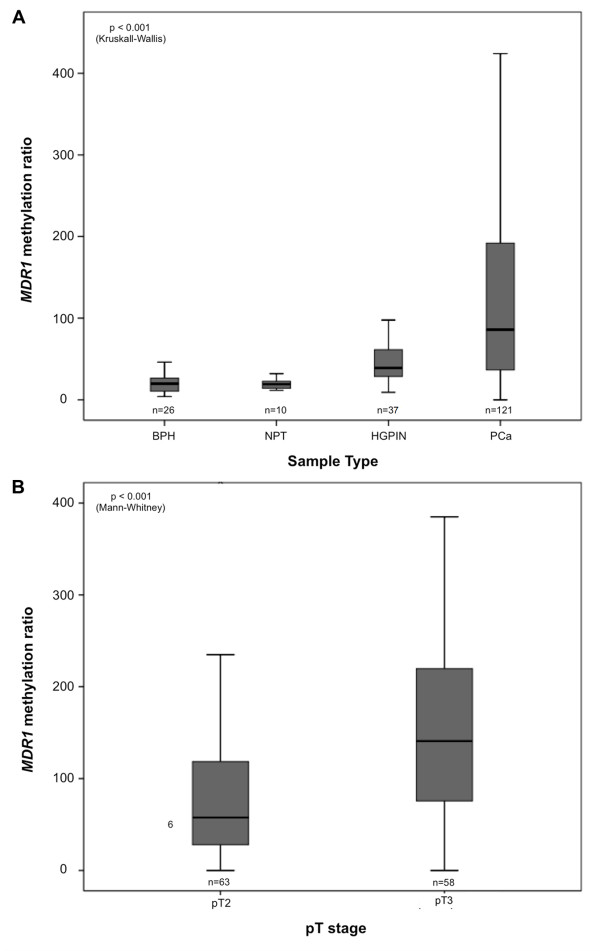
**Distribution of *MDR1 *methylation levels among prostatic tissues.** [**(A)** benign prostatic hyperplasia (BPH), morphologic normal prostatic tissue (NPT), high-grade prostatic intraepithelial neoplasia (HGPIN), and prostate cancer (PCa)], and in prostate cancer samples according to the pathological stage **(B)**.

Overall, the highest *MDR1* methylation frequencies and levels were found in PCa cases, whereas NPT disclosed the lowest levels (Table 2 and Figure 1A). The Kruskall-Wallis test detected significant differences in methylation levels among the four groups of samples (p < 0.001). Pair-wise comparisons showed that *MDR1* methylation levels in PCa were significantly higher than those of HGPIN, BPH and NPT (Mann–Whitney, Bonferroni- adjusted, p < 0.001 for all comparisons). Moreover, *MDR1* methylation levels were statistically similar between BPH and NPT, but both were significantly lower than those of HGPIN (Mann–Whitney U, Bonferroni-adjusted, p < 0.001 for both). Interestingly, locally invasive PCa cases displayed higher *MDR1* methylation levels than organ-confined tumors (Mann–Whitney, p < 0.001) (Figure 1B). No association was found with Gleason score (Kruskall-Wallis, p = 0.097), age or serum PSA (Spearman’s correlation test, r_s_ = -0.10, p = 0.203 and r_s_ = 0.09, p = 0.297, respectively).

**Table 2:** **Frequency of positive cases for *MDR1 *** **promoter methylation, distribution of methylation levels in normal prostate tissue and prostate lesions [Median (IQR: interquartile range)], and correlation with histopathological parameters**

	**n**	**Frequency (%)**	***p-*** value*	**Median (IQR)**	*p*-value**
**Tissue sample**			p < 0.001		p < 0.001
**BPH**	26	11.5 (3/26)		19.8 (11.9-26.8)	
**NPT**	10	0 (0/10)		19.0 (14.3–21.9)	
**HGPIN**	37	37.8 (14/37)		38.6 (28.3–59.2)	
**PCa**	121	67.8 (82/121)		85.8 (36.6 - 192.0)	
**Gleason score**			p = 0.142		p = 0.097
***<7***	54	63.0 (34/54)		67.7 (29.4-158.2)	
**7**	60	68.3 (41/60)		115.1 (37.6-227.7)	
***>7***	7	100.0 (7/7)		85.8 (75.6-195.6)	
**Tumor stage**					p < 0.001
**pT2**	63	54.0 (34/63)	p = 0.001	57.6 (29.4 - 158.2)	
**pT3**	58	82.8 (48/58)		140.8 (37.6 - 227.7)	

*Chi-square test for trend, **Kruskall–Wallis ANOVA or Mann–Whitney test, as appropriate.

### P-gp immunoexpression in prostatic tissues

**Figure 2:**
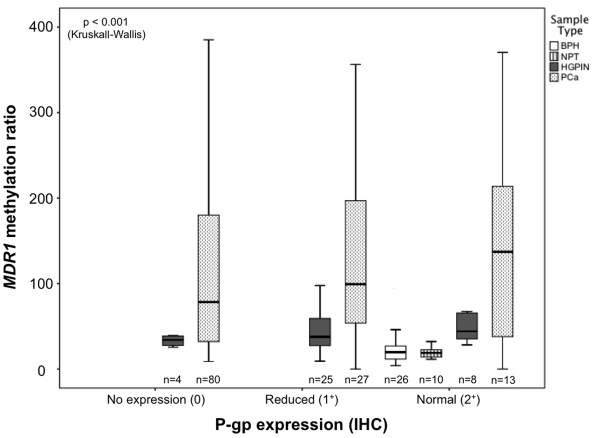
**Distribution of *MDR1 *** **methylation levels in prostate tissues [benign prostatic hyperplasia (BPH), morphologic normal prostatic tissue (NPT), high-grade prostatic intraepithelial neoplasia (HGPIN), and prostate cancer (PCa)] according with P-gp protein expression categorization [2**^**+**^**(expression similar to normal prostate tissue), 1**^**+**^**(expression lower than normal prostate tissue), and 0 (no immunoexpression)].**

As expected, immunoreactivity for P-gp was found in the cell membrane and cytoplasm. Statistically significant differences in P-gp expression among the four groups of samples were detected (Chi-square test, p < 0.001). Most PCa (89.3%) and HGPIN (81.1%) samples showed decreased P-gp expression (scores 0 and 1^+^), while all BPH and NPT exhibited normal expression (2^+^). Thus, a decrease in immunoexpression of P-gp was apparent from non-tumorous prostate tissues, to HGPIN, to tumors (Somers’d = -0.702, p < 0.001) (Table 3). Pairwise comparisons disclosed significant differences in all cases (Mann–Whitney, Bonferroni-adjusted, p < 0.001) except for NPT *vs.* HBP. A representative example of P-gp immunoexpression results is provided in Figure 2B.

**Table 3:** P-gp immunoexpression in prostatic tissues

	**Scoring**				
	**n**	**0**	**1**^**+**^	**2**^**+**^	**Somers’d,** ***p-value***
NPT	10	0	0	10 (100)	-0.702, *p* < 0.001
BPH	26	0	0	26 (100)	
HGPIN	37	5 (13.5)	25 (67.6)	7 (18.9)	
PCa	121	81 (66.9)	27 (22.3)	13 (10.8)	

NPT- morphologically normal prostatic tissue; BPH- benign prostatic hyperplasia; HGPIN- high grade prostatic intraepithelial neoplasia, PCa - prostate carcinoma.

### P-gp immunoexpression and MDR1 promoter methylation in prostate tissues

The distribution of *MDR1* methylation levels in prostate tissues according to P-gp immunoexpression is graphically displayed in Figure 2. Statistical analysis demonstrated significant differences in methylation levels among immunoexpression groups 0 and 2^+^, and 1^+^ and 2^+^ (Mann–Whitney, Bonferroni-adjusted p < 0.001 for both), when all types of prostate tissue samples were considered. However, no statistically significant differences in *MDR1* methylation levels were apparent between tumors scored 0 and 1^+^ for P-gp immunoexpression. Thus, the differences depicted between immunoexpression groups 0 and 1^+^, in the one hand, and group 2^+^, in the other, are mainly due to the inclusion of non-cancerous tissues, i.e., BPH and NPT in group 2^+^.

### Methylation and expression analysis of MDR1 in PCa cell lines

To further assess whether *MDR1* was epigenetically deregulated in PCa, the four cell lines were exposed to epigenetic modulating drugs and the results were analyzed either by bisulfite sequencing, qMSP or qRT-PCR.

**Figure 3:**
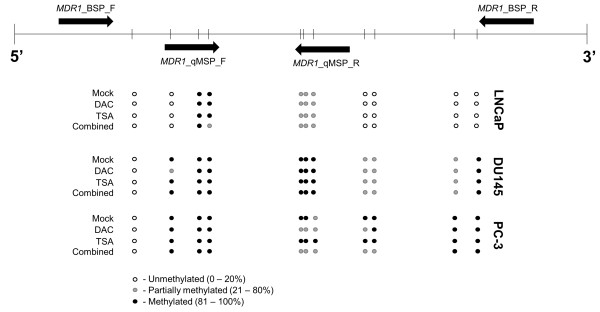
**Characterization of the methylation status of individual CpG dinucleotides by bisulfite sequencing.** The upper panel of the diagram represents the region of the gene under analysis, bisulfite sequence (BSP) and quantitative MSP (qMSP) primers (black arrows) and CpG dinucleotides density (vertical bars). The lower panel shows the status of methylation for each CpG dinucleotide for three different PCa cell lines (LNCaP, DU145 and PC3) exposed to epigenetic drugs. White circle – unmethylated CpG (0–0.20); grey circle – partially methylated CpG (0.21 - 0.80); black circle – fully methylated CpG (0.81 - 1.0).

Bisulfite sequencing was performed in three PCa cell lines – LNCaP, DU145 and PC3 - to specifically assess the methylation status of eleven CpG dinucleotides localized in the analyzed promoter region, before and after exposure to DAC and/or TSA (Figure 3). According to the results, LNCaP was the one with the lower number of methylated sites, whereas PC3 displayed the higher number of methylated CpG dinucleotides. After treatment with epigenetic modulating drugs, no significant changes were observed, except for PC3, which upon exposure to DAC, either alone or combined with TSA, displayed partial loss of methylation at some CpG.

**Figure 4:**
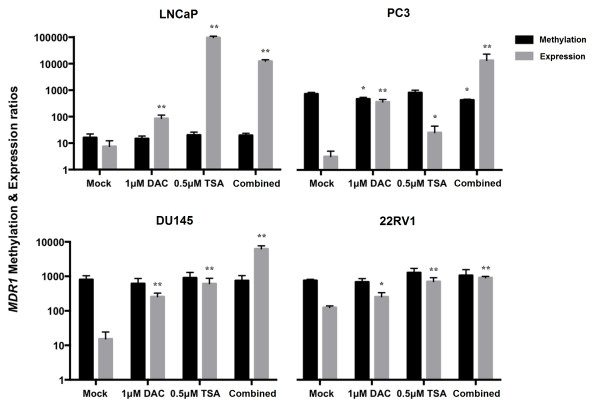
**Methylation and expression levels of *MDR1 *in prostate cancer cell lines LNCaP, PC3, DU145 and 22RV1 in both untreated (Mock) and DAC or/and TSA treated cells.** Asterisks represent statistically significant differences comparing to Mock, *p < 0.05; **p < 0.001. The represented scale is logarithmic.

Additionally, methylation levels of all prostate cancer cell lines (LNCaP, 22Rv1, DU145 and PC3) were also tested by qMSP in a smaller region comprised within the sequence analyzed by bisulfite sequencing (Figure 4). All PCa cells displayed methylation at the DSP promoter region of *MDR1*, although levels were variable. The highest methylation levels were observed for PC3, which concurrently displayed the lowest mRNA relative expression levels, corroborating bisulfite sequencing results. In addition, as previously observed, LNCaP cells were those depicting the lowest methylation levels. Furthermore, and except for PC3 in which a slightly significant decrease in methylation levels was detected after treatment with DAC alone or combined with TSA (Dunnet’s test, p = 0.027 and p = 0.012, respectively), no significant effects were found in methylation levels compared to mock cells in PCa cell lines, in line with bisulfite sequencing analysis results.

Nevertheless, *MDR1* expression levels increased in all cancer cell lines after treatment with epigenetic modulating drugs (Dunnet’s test, 0.001 ≤ p ≤ 0.015). Interestingly, re-expression levels were significantly higher when DAC and TSA were used in combination, in all cell lines (Dunnet’s test, p < 0.001) except for LNCaP, in which TSA alone induced the most impressive enhancement in *MDR1* transcript levels (Dunnet’s test, p < 0.001).

### Effect of epigenetic modulating drugs on activating histone marks at the MDR1 promoter in PCa cell lines

Because histone post-translational modifications are also associated with gene transcription activation/repression status, ChIP analysis was carried out for the native *MDR1* promoter in LNCaP, DU145 and PC3 cell lines, after treatment with TSA alone or combined with DAC. Moreover, since the effect in *MDR1* re-expression of DAC alone was modest, the histone marks were not assessed for this treatment regimen.

**Figure 5:**
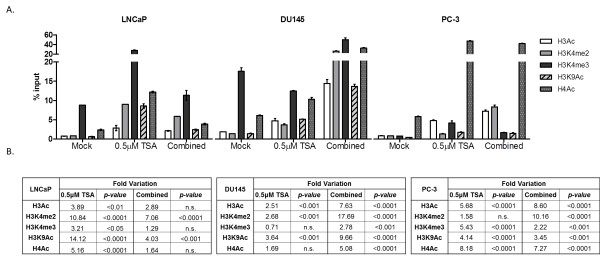
**ChIP analysis of transcriptionally activating histone marks (H3Ac; H3K4me2; H3K4me3; H3K9Ac; H4Ac) binding to *MDR1 *promoter in LNCaP and DU145 cell lines both in untreated (Mock), TSA and TSA combined with DAC treated cells. (A)** ChIP-qPCR data, normalized to the percent input method. **(B)** Fold variation of each histone activating mark, comparing treated cells with Mock cells.

**Figure 6:**
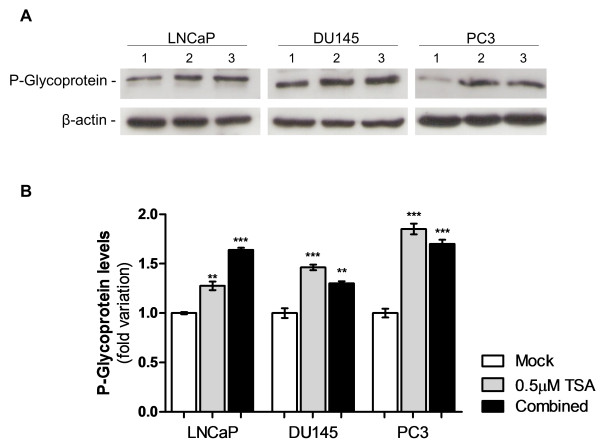
**Effect of epigenetic drugs in P-glycoprotein expression in PCa cell lines after pharmacological treatment.(A)** Protein gel blot analysis (1- Mock; 2–0.5 μM TSA; 3- 1μM DAC + 0.5 μM TSA). β-actin was used as loading control. **(B)** Relative levels of Pglycoprotein. Values of three independent experiments (average ± standard deviation) are represented as fold variation in comparison to Mock cells. Optical density of visible bands was determined and normalized with β-actin levels (**p < 0.01, ***p < 0.001; Oneway ANOVA, Dunnett’s Multiple Comparison Test).

Interestingly, for all cell lines, enrichment in histone activating marks (H3Ac, H3K4me2, H3K4me3, H3K9Ac and H4Ac) at the *MDR1* gene promoter was found, after exposure to TSA alone or in combination with DAC, compared to untreated cells (Figure 5). The active marks’ fold variation differed among the treatments and respective cell lines, in accordance with the above mentioned re-expression data, excluding PC3. Whereas in LNCaP cells TSA exposure induced an impressive increase in the accumulation of all the studied activation marks within *MDR1* promoter, in DU145 the most evident effect was observed after combined treatment. However, no obvious increase in fold of the assessed activating marks was observed in PC3 cells for both treatments. Among those marks, H3K4me2 was the one which showed the highest variation in all cell lines. Collectively, these findings indicate that *MDR1* expression is mostly regulated through histone post-translational covalent modifications. Indeed, western blot results showed that P-glycoprotein levels increased after exposure to TSA alone (p < 0.01 for LNCaP and p < 0.001 for DU145 and PC3) or in combination with DAC (p < 0.001 for LNCaP and PC3 and p < 0.01 for DU145) (Figure 6).

## Discussion

The P-glycoprotein, encoded by the *MDR1*/*ABCB1* gene, is a transmembrane protein involved in ATP-dependent transport of specific substrates across lipid membranes, playing an important role in steroid metabolism and in the export of metabolites, carcinogens and cytotoxic drugs such as anthracyclines, taxanes, vinca alkaloids and epipodophyllotoxins [2,4]. Although several mutations in this transporter have been associated with human disease, the actual consequences in its function are still controversial [4]. In PCa, *MDR1* is frequently methylated at its promoter region (an epigenetic event mostly associated with gene silencing) compared to non-tumorous prostate tissues and has thus been proposed as a PCa biomarker [11-13]. Although a few studies have correlated *MDR1* promoter methylation with decreased transcription in PCa [11], the biologic impact of this epigenetic alteration and its role in prostate carcinogenesis has not been fully elucidated. Herein, we showed that although *MDR1* promoter methylation is, indeed, a frequent event in PCa, occurring early in prostate carcinogenesis and it is associated with decreased mRNA and protein levels. However, promoter methylation does not seem to be prime cause of *MDR1* silencing. Instead, our results indicate that post-translational histone modifications constitute the mechanism underlying *MDR1* deregulation in PCa.

Promoter methylation of several cancer-related genes (e.g., *GSTP1*, *APC*, *RARB2*, and *RASSF1A*) has been extensively documented in PCa and seems to occur early in prostate carcinogenesis, as demonstrated by its intermediate frequency in HGPIN lesions [16]. High frequency of *MDR1* promoter methylation has been previously reported in PCa, ranging between 54% and 88% [11-13]. In our study, *MDR1* methylation was found in 67.8% of the tumors, which is within the range of previous reports, as well as in 37.8% of HGPIN lesions. To the best of our knowledge, our study is the first to demonstrate *MDR1* methylation in HGPIN lesions, which are precursors of prostate adenocarcinoma. Moreover, non-cancerous prostate tissues, including NPT and HBP, were scarcely methylated, in line with previous reports [11,12]. Taken together, these results sustain that the progressive methylation of genes that play critical roles in controlling cell proliferation or differentiation, as well as protection of DNA from mutagens, constitutes an important mechanism not only in neoplastic transformation, but also in the process of tumor progression in the prostate. This postulate is further supported by the association between methylation levels of several of the aforementioned genes with tumor aggressiveness [23,24]. Additionally, we found that *MDR1* promoter methylation levels increased with pathological stage, in accordance with previous studies, which also disclosed an association with higher Gleason score [13], not found in our series. Thus, *MDR1* seems to follow the same trend of other cancer-related genes in prostate carcinogenesis.

The association of aberrant promoter methylation with gene silencing has been demonstrated for several genes in many cancer models [16,25,26]. In this study, we found that the expression of P-gp, the protein encoded by *MDR1*, was inversely correlated with promoter methylation levels. Similar results were previously reported for PCa at protein [27] and transcript level [11]. Thus, it seems reasonable to assume that aberrant CpG island methylation was responsible for *MDR1* silencing in PCa. To test that hypothesis, we exposed four PCa cell lines to epigenetic modulating drugs, which are able to reverse DNA methylation and histone deacetylation. Surprisingly, increased expression of *MDR1* mRNA was not associated with a decrease in methylation levels, but also the highest re-expression levels were observed when the demethylating agent was associated with the HDAC inhibitor. These results strongly suggested that histone modifications are more likely to be the main cause of *MDR1* silencing in PCa. It could be argued that the concentration of DAC to which PCa cell lines were exposed was not sufficient to induce *MDR1* demethylation. However, not only the same concentration is effective for inducing demethylation of other genes in PCa cell lines [28,29] but also the use of TSA alone was able to increase *MDR1* expression in our experiments. Nevertheless, *MDR1* expression was more robustly up-regulated following exposure to both epigenetic modulating drugs. It might be questioned whether the analyzed region of the *MDR1* gene is critical for regulation of expression. Nonetheless, it has been previously demonstrated that the CpG island analyzed in our study was involved in the regulation of *MDR1* transcriptional activity [4,6-8] and that it was densely methylated in PCa [13]. Alternatively, the possibility that exposure to DAC and TSA leads to re-activation of genes which positively regulate *MDR1* can not be dismissed.

To further investigate whether histone modification might be involved in *MDR1* silencing in PCa, we compared histone active marks (H3Ac, H3K4me2, H3K4me3, H3K9Ac and H4Ac) at the *MDR1* gene promoter, after exposure to TSA alone or in combination with DAC, compared to untreated cells. We found an overall enhancement in these active marks following exposure to TSA and/or DAC, in particular the H3K4me2 mark. Importantly, these findings were correlated, at protein level, with an increase in P-gp expression. Interestingly, enhancement of H3Ac at the *MDR1* promoter has been previously correlated with gene activation in sarcoma cell lines [30]. Thus, our results indicate that histone onco-modifications are likely to be the most important epigenetic event associated with *MDR1* downregulation in PCa, although it is associated with dense CpG island methylation. There is some controversy regarding which epigenetic alteration arises first and how it relates to effective gene silencing. Remarkably, in PCa, histone onco-modifications herald CpG methylation in *RASSF1A* downregulation whereas the opposite occurs for *GSTP1* inactivation [31,32]. Although either scenario might fit our observations, the occurrence of promoter methylation early in prostate carcinogenesis (HGPIN) and the experimental gene re-expression observed following exposure to epigenetic modulating drugs without significant changes in promoter methylation levels, suggest that CpG methylation precedes histone onco-modifications.

*MDR1* has been associated with the “multidrug resistance” phenotype [4]. Thus, from a biological standpoint, it is almost counterintuitive that *MDR1* gene silencing is associated with PCa development and progression as the loss of P-gp expression may be interpreted as an unfavorable change for neoplastic cells. However, even in other tumor models, P-pg expression was found to be higher in localized than in metastatic disease [33] also indicating a connection between P-gp loss of expression and tumor progression. This is also supported by the previous finding that *MDR1* downregulation was associated with increased cell proliferation and unaltered apoptosis in PCa [11]. Nevertheless, *MDR1* silencing may also provide a therapeutic opportunity PCa treatment. Owing to the role of this protein in the removal of xenobiotics (e.g., taxanes, Vinca alkaloids) from the intracellular milieu/environment into urine and bile, thus promoting their elimination [4] decreased expression of P-gp is likely to increase the efficacy of chemotherapeutic agents for treatment of cancer patients. This assumption may be of clinical relevance, since castration-resistant PCa patients treated with regimens that include taxanes have improved survival rates [34]. It is, then, tempting to speculate whether *MDR1* promoter methylation and/or P-gp expression might constitute biomarkers predictive of response to taxane therapy in PCa patients.

## Conclusion

In conclusion, we have shown that *MDR1* aberrant promoter methylation and decreased expression are common events in PCa. These alterations seem to occur early in prostate carcinogenesis and promoter methylation is associated with clinicopathological features of tumor aggressiveness. Although *MDR1* promoter methylation is inversely correlated with gene expression, effective *MDR1* silencing is mostly likely due to histone onco-modifications, which may be heralded by CpG methylation at regulatory sites.

## Methods

### Patients and samples

Tissue samples of PCa were collected from 121 patients consecutively diagnosed and primarily treated with radical prostatectomy at Portuguese Oncology Institute – Porto. In 37 cases, a dominant HGPIN lesion was identified and collected for further analysis. BPH specimens were collected from 26 patients submitted to transurethral resection of the prostate and 10 NPT were procured from the peripheral zone of prostates that did not harbor PCa (obtained from cystoprostatectomy specimens of bladder cancer patients) and these were used as controls. All specimens were fresh-frozen at -80ºC and subsequently cut in a cryostat for microscopic evaluation and selection of areas for analysis. Cut sections were trimmed to maximize target cell content (>70%). From each specimen, parallel fragments were collected, formalin-fixed and paraffin-embedded for routine histopathological examination, including Gleason scoring [35] and pathological staging [36], by an expert pathologist. Relevant clinical data was collected from the clinical charts. This study and respective informed consent from was approved by the institutional review board [Comissão de Ética para a Saúde-(IRB-CES-IPOFG-EPE 019/08)] of Portuguese Oncology Institute - Porto, Portugal.

### Cell culture and treatment with epigenetic modulating drugs

To assess the role of epigenetic mechanisms in *MDR1* altered expression, representative PCa cell lines DU145 [obtained from the American Type Culture Collection (ATCC, Lockville, MD)], LNCaP and PC3 (kindly provided by Prof. Ragnhild A. Lothe from the Department of Cancer Prevention at The Institute for Cancer Research, Oslo, Norway) and 22Rv1 (kindly provided by Dr. David Sidransky at the Johns Hopkins University School of Medicine, Baltimore, MD, USA) were exposed to epigenetic modulating drugs. Cell lines were cultured according to the manufacturer’s specifications, with 10% fetal bovine serum (Gibco, USA) and antibiotics (100 units/mL penicillin G, and 100 μg/mL streptomycin, Gibco), in a humidified atmosphere of 5% CO_2_ at 37ºC. All PCa cell lines were karyotyped by G-banding (for validation purposes) and routinely tested for Mycoplasma spp. contamination (PCR Mycoplasma Detection Set, Clontech Laboratories, USA). The four cell lines were grown and treated with a pharmacologic inhibitor of DNA methyltransferases DAC (Sigma-Aldrich®, Germany) - 1 μM for 72 h - and/or a pan-inhibitor of histone deacetylases TSA (Sigma-Aldrich®, Germany) - 0.5 μM - added in the final 24 h. In parallel, the same cell lines were cultured without treatment for 72 hours and were harvested before confluence. The medium and the drugs were changed every 24 h. After 72 hours, cells were harvested, by tripsinization, for DNA and RNA extraction or fixed and scraped for Chromatin Immunoprecipitation (ChIP) assays. Protein extracts were also obtained using RIPA lysis buffer (Santa Cruz Biotechnology, CA, USA).

### Isolation of nucleic acids and bisulfite treatment

DNA from prostate tissues and cell lines was extracted by the phenol-chloroform method, at pH 8, as described by Pearson *et al.*[37]. Total RNA from tissue samples and cancer cell lines was isolated using Trizol (Invitrogen, USA). DNA and RNA concentrations were determined using a ND-1000 Nanodrop (NanoDrop Technologies, USA).

All DNA samples were submitted to sodium bisulfite modification, based on the previously described method [38,39]. Briefly, 2 μg of genomic DNA from each sample were used for the chemical treatment. Bisulfite-modified DNA was purified using a vacuum manifold and a Wizard DNA Clean-up System [Promega Corporation, USA], treated again with sodium hydroxide, precipitated with ethanol, eluted in 120 μl of water and stored at -80ºC.

### Bisulfite sequencing

Bisulfite modified DNA from three different PCa cell lines (LNCaP, DU145 and PC3), exposed to DAC and/or TSA as abovementioned, was used to evaluate the methylation status of CG dinucleotides, by bisulfite sequencing using primers for a specific sequence of *MDR1* promoter, addressing the same region that was further analyzed by qMSP [11]. The protocol was performed as described elsewhere [40]. PCR reactions for direct included a 10-minute 94ºC denaturation step followed by 40 cycles of 94ºC for 30 seconds, annealing temperature for 30 seconds, and 72ºC for 30 seconds. PCR products were loaded onto a nondenaturing 2% agarose gels, stained with ethidium bromide and visualized under an ultraviolet transilluminator. Excess primer and nucleotides were removed by Illustra GFX PCR DNA and Gel Band Purification kit (GE Healthcare, USB Corporation, Cleveland, OH) following the protocol of the manufacturer. The purified products were sequenced using the dGTP BigDye Terminator Cycle Sequencing Ready Reaction kit (Applied Biosystems) in an ABI PRISMTM 310 Genetic Analyzer (Applied Biosystems). The approximate amount of methyl cytosine of each CpG site was calculated by comparing the peak height of the cytosine signal with the sum of the cytosine and thymine peak height signals [41]. CpG sites with ratio ranges 0–0.20, 0.21-0.80, and 0.81-1.0 were considered unmethylated, partially methylated, and fully methylated, respectively.

### Quantitative methylation-specific PCR (qMSP)

The modified DNA was used as a template for real-time fluorogenic qMSP. All samples were subjected to two reactions of amplification, one for the quantification of methylated *MDR1* and the other for quantification of an internal reference gene (*β-actin*) using primers and probes reported elsewhere [11,23]. The converted DNA, positive and negative controls, and commercial standards with serial dilutions of fully methylated DNA were amplified in the same run. These standards were used to construct a calibration curve in order to quantify the fully methylated genes in the two reactions. Fluorogenic qMSP assays were carried out in an Applied Biosystems 7000 Sequence Detector (Applied Biosystems, USA). PCR was performed in separate wells for each primer/probe set and each sample was run in triplicate. The final reaction mixture consisted of 600 nM of each primer (Invitrogen, USA); 200 nM probe (Applied Biosystems, USA); 0.75 unit of platinum Taq polymerase (Invitrogen, USA); 200 μM each of dATP, dCTP, dGTP, and dTTP; 16.6 mM ammonium sulfate; 67 mM Trizma; 6.7 mM magnesium chloride; 10 mM mercaptoethanol; 0.1% DMSO, and 3 μL bisulfite-converted genomic DNA. PCR was performed using the following conditions: 95ºC for 2 min, followed by 50 cycles at 95ºC for 15 s and 60ºC for 1 min. For each sample, the relative level of methylation in *MDR1* promoter was obtained by dividing the value of methylated *MDR1* by the respective value of *β-actin*, which was then multiplied by 1000 for easier tabulation.

### Quantitative reverse-transcription PCR (RT-qPCR)

Total RNA from all PCa cancer cell lines untreated, treated either with 1 μM of DAC for 72 hours, or treated with the combination of 1 μM of DAC (72 h) and 0.5 μM of TSA (added in the last 12 h) was analyzed. From each sample, 0.5 μg of total RNA was transcribed into cDNA by reverse transcription using the RevertAidTM H Minus First Strand cDNA Synthesis Kit (Fermentas, Canada), including random hexamer primers. The cDNA was used as the template for the real-time quantitative PCR reaction. *MDR1* (Hs01070651_m1, Applied Biosystems, USA), and the endogenous control assay GUSB (Hs99999908_m1, Applied Biosystems, USA) were amplified separately in 96-well plates following the recommended protocol (Applied Biosystems, USA), and the real time quantitative gene expression was measured by the 7500 Real-Time PCR System (Applied Biosystems, USA). All samples were analyzed in triplicate, and the mean value was used for data analysis. The human universal reference RNA (Stratagene, USA) was used to generate a standard curve on each plate, and the resulting quantitative expression levels of the tested gene were normalized against the mean value of the endogenous control to obtain a ratio that was then multiplied by 1000 for easier tabulation.

### Immunohistochemistry

Immunohistochemistry was performed according to the avidin-biotin method using the VECTASTAIN® Universal Elite ABC Kit [©Vector Laboratories, USA]. Sections (3 μm thick) from paraffin-embedded tissues, corresponding to the samples used for methylation analysis, were deparaffinised in xylene and hydrated through a graded alcohol series. Antigen retrieval was accomplished by microwaving the specimens at 800 W for 5 minutes in EDTA buffer. After cooling the slides, endogenous peroxidase activity was blocked by incubating the sections in hydrogen peroxide in 3% methanol for 30 minutes. The sections were treated with 5% normal horse serum in 1% PBS-BSA for 30 minutes to reduce background interference. The primary mouse monoclonal antibody (C494 clone, Thermo Scientific, UK) was applied in 1:50 dilution with 1% PBS-BSA and left at 4ºC overnight. The secondary biotinylated horse antibody at a dilution of 1:50 was added for 30 minutes. To enhance the immunohistochemical staining, sections were incubated in avidin-biotin complexes for 30 minutes. Then, 3,3′-diaminobenzidine (Sigma-Aldrich®, Germany) was used for visualization and hematoxilin for nuclear counterstaing. Finally, after dehydration and diaphanization, slides were mounted in Entellan® (Merck-Millipore, Germany). Adrenal gland tissue sections showing intense immunoreactivity for P-gp, were used as positive controls. The negative control consisted on the omission of the primary antibody. Assessment of antibody expression was performed by a pathologist (RH) blinded to molecular analyses data. Imunohistochemistry results were categorized according to stain intensity as 2^+^ (expression similar to normal prostate tissue), 1^+^ (expression lower than that of normal prostate tissue), and 0 (no immunoexpression).

### Chromatin immunoprecipitation (ChIP) Assay

EZ-Magna ChIP™ G-Chromatin Immunoprecipitation Kit and the Magna Grip™ Rack (Merck-Millipore, Germany) were used to perform ChIP assay according to the manufacturer’s instruction. For each chromatin immunoprecipitation, 5 μg of anti-AcH3 (06–599, Millipore, USA), anti-H3K4me2 (ab32356, Abcam, UK), anti-H3K4me3 (ab8580, Abcam, UK), anti-AcH3K9 (17–658, Millipore, USA), anti-AcH4 (06–866, Millipore, USA) and 1 μL of the negative control provided with the kit (normal mouse IgG) were used. Quantification of DNA was performed in a 7000 Real-Time PCR System (Applied Biosystems, USA), using Power SYBR® Green PCR Master Mix (Applied Biosystems, USA) and gene-specific primers for gene promoter of *MDR1* – sense: 5′-AGTCATCTGTGGTGAGGCTGAT-3′; anti-sense: 5′-TACTCGAATGAGCTCAGGCTTC-3′ [≈ 800 bp upstream Transcription Start Site (TSS)]. The relative amount of promoter DNA was normalized using Input Percent Method.

### Western blot

For mock and treated LNCaP, DU145 and PC3 cell lines, protein extract concentrations were determined using Pierce® BCA Protein Assay Kit (Thermo Scientific, Inc., Bremen, Germany). Subsequently, 30 μg of total protein were loaded in each well, and separated by SDS-PAGE, transferred to nitrocellulose membranes and probed with antibodies against P-glycoprotein (Abcam, ab129450 at 1:1000) or the endogenous control β-actin (Sigma-Aldrich, A1978 at 1:10000). Secondary antibodies, conjugated with horseradish peroxidase, were incubated at a dilution of 1:3000. Finally, blots were developed using Immun-Star™ WesternC™ Kit according to manufacturer’s indications (BioRad, Hercules, CA, USA) and exposed to Amersham Hyperfilm (GE Healthcare). Experiments were done with biological triplicates. Relative optical density determination was performed using QuantityOne® Software version 4.6.6. (Biorad, Hercules, CA, USA).

### Statistical analysis

As the analyzed variables did not follow a normal distribution, nonparametric tests were used. In each group of samples, frequencies of *MDR1* methylation were compared using the Chi-square test for trend. Median and interquartile range (p25-p75) of *MDR1* methylation levels were also determined, and then compared using Kruskall-Wallis test or the Mann–Whitney U-test, depending on the number of categories in each group. Likewise, the relationship between methylation ratios and standard clinicopathological variables (age, serum PSA levels, tumor grade and stage), were evaluated using the Kruskall-Wallis or Mann–Whitney tests. A Spearman nonparametric correlation test was additionally performed to compare age and methylation levels.

Frequencies of immunoexpression along sample groups were compared using the Chi-square test, and the directional measure Somers’d was additionally computed. Somers’ statistic varies from -1 to 1 and assesses the association between two ordinal variables, with a value of 1 indicating a strong positive association, and a value of -1 indicating a strong negative one. The correlation between *MDR1* promoter methylation levels and P-gp immunoexpression was assessed using the Kruskall-Wallis test, followed by Mann–Whitney U-tests. For this analysis, all sample types were considered within the same group of immunoexpression score.

In cell lines, differences in transcript and methylation levels between treatments were determined using One-Way Analysis of Variance (one-Way ANOVA) test, followed by a multiple comparisons Dunnet’s test, comparing all groups against the Mock. Differences regarding protein levels were also evaluated using a one-Way ANOVA test, followed by a multiple comparison Dunnet’s test, comparing all groups against the experimental control.

All tests were two-sided and p-values were considered significant when inferior to 0.05. For multiple comparisons the Bonferroni method was used to adjust for p values. Statistical analyses were performed using a computer-assisted program (SPSS version 20.0, USA).

## Competing interests

The authors declare that they have no competing interests.

## Authors’ contributions

RH, AIO and CJ wrote the manuscript with input from co-authors. RH is an experienced pathologist and greatly contributed for tumors classification and immunohistochemistry analysis. ATM was responsible for preparation of formalin fixed, paraffin-embedded tissues for immunochemistry analysis. AIO and VLC carried out the DNA and RNA extraction from clinical samples, and the methylation and expression analysis. AIO and TB performed cell culture and drug exposure assays. AIO carried out ChIP assays. TB executed Western Blot and direct bisulfite sequencing analysis. AM and JO were responsible for clinical data collection from patients’ records. Statistical analysis was performed by RH, AIO, CJ and TB. CJ was responsible for the experimental design and management of the project. All authors read and approved the final manuscript.
